# Eco-friendly fabrication of bacterial cellulose nanopaper with enhanced strength and toughness from a powder suspension

**DOI:** 10.3389/fbioe.2026.1923616

**Published:** 2026-08-25

**Authors:** Dieter Rahmadiawan, Hairul Abral, Muhammad Irfan, Fadli Hafizulhaq, Sundaravadanam Vishnu Vadanan, Sierin Lim, Shih-Chen Shi

**Affiliations:** 1 Department of Mechanical Engineering, National Cheng Kung University (NCKU), Tainan, Taiwan; 2 Department of Mechanical Engineering, Universitas Negeri Padang, Padang, Sumatera Barat, Indonesia; 3 Laboratory of Nanoscience and Technology, Department of Mechanical Engineering, Andalas University, Padang, Indonesia; 4 School of Chemistry, Chemical Engineering and Biotechnology, Nanyang Technological University, Singapore, Singapore

**Keywords:** bacterial cellulose, boiling water treatment, crystallinity, toughness, ultrasonication

## Abstract

The growing demand for sustainable alternatives to petroleum-derived materials in packaging, electronics, and filtration highlights the need for robust and eco-friendly fabrication methods for cellulose-based films. However, cellulose nanopapers with improved mechanical performance are commonly produced using chemical modification or energy-intensive processing, which may limit their scalability and environmental sustainability. In this study, bacterial cellulose (BC) nanopapers with enhanced strength and toughness were fabricated from a non-oxidized BC powder suspension using ultrasonication followed by boiling-water (BW) treatment. BC powder was prepared from wet BC pellicles hydrated in distilled water, sonicated, boiled at 100 °C for 2, 4, and 5 h, and oven-dried. Nanopapers were then fabricated by resuspending the treated BC powder in water followed by casting. The results showed that BW treatment simultaneously improved tensile strength and toughness. The 5-h boiling treatment results in increased tensile strength, tensile modulus, elongation at break, and toughness by 89% (81.9 MPa), 261% (1,383.5 MPa), 26% (14.8%), and 148% (6.7 MJ/m^3^), respectively, compared with the non-boiled nanopaper. The BW-treated nanopaper also exhibited a broader plastic deformation region and fewer pores. These findings demonstrate that boiling-water treatment of an ultrasonicated, non-oxidized BC powder suspension provides a simple, chemical-free, and pressure-free route for producing BC nanopapers with enhanced mechanical performance.

## 1 Introduction

As global industries increasingly seek sustainable alternatives to petroleum-based materials (Sandra et al., 2025; Rahmadiawan et al., 2022; Nurdin et al., 2025; Fauza et al., 2023; Rahmadiawan et al., 2021a), bio-derived polymers have emerged as promising candidates for next-generation functional materials (Huang et al., 2024; Rahmadiawan et al., 2021b; Kadriadi et al., 2025). Among them, cellulose has gained widespread attention due to its abundance and tunable properties. Cellulose from renewable sources has remarkable properties, including biodegradability, natural abundance, excellent mechanical and thermal properties, low density, non-toxic, and low cost (Wu et al., 2023; Rahmadiawan et al., 2025; Yulianti et al., 2024; Sharif et al., 2021). These advantages have been attracting attention to explore the application of cellulose as the base material for the fabrication of nanopapers (Wang et al., 2018a) and green membranes (Voicu et al., 2016; Sheng et al., 2020). However, the fabrication of the nanopaper is typically carried out using cellulose nanofibers (CNFs) extracted from wood using chemical treatment (Xu et al., 2016; Huang, 2018).

BC pellicle with high cellulose purity, not bound with other chemicals, has been explored as an alternative source of CNFs (Abdul Khalil et al., 2020; Gea et al., 2011). BC pellicle can be produced from *Gluconacetobacter xylinum* bacteria due to its high productivity (Liu et al., 2020). BC nanofibers isolated from the BC pellicle have excellent mechanical properties, high crystallinity, thermal resistance and stability, and good compatibility (Hosseini et al., 2018; Yagyu et al., 2015). Intriguingly, it can also be applied in various applications, such as lubricant additives (Fuadi et al., 2022; Sakakibara et al., 2021), reinforcing materials via self-aggregated lamination (Li et al., 2020), and oil-water separation membranes with super-wettability (Sai et al., 2020).

Previous work successfully mass-produced BC nanofibers using a series of 30-L fermentation reactors (Gwon et al., 2019). The fabrication process is more environmentally friendly than those isolated from plant fibers (Rozenberga et al., 2016). Furthermore, BC has also been used as a natural binder to develop recyclable wood-based composites without chemical delignification (Fang et al., 2022). However, a fibrous network of three-dimensional structures and the high water content of native BC pellicles limit their applications (Pham et al., 2020). Therefore, we propose that wet native BC pellicles can be processed into dry BC powder to expand their application potential in many areas.

Cellulose nanopaper is a product made entirely of CNFs (Yousefi et al., 2013). However, producing strong and tough cellulose nanopaper from BC powder remains challenging (Meng and Wang, 2019; Zhu et al., 2015). Its mechanical and thermal properties are strongly influenced by fiber network density and porosity (Wang et al., 2018a). Porous cellulose paper generally shows lower tensile strength and thermal resistance than dense paper (Henriksson et al., 2008).

Ultrasonication has been used to improve the dispersion of nanocellulose suspensions before film casting. Csiszár et al. reported that low-frequency ultrasonication reduced cellulose aggregate size and progressively increased the tensile strength of cast nanocellulose films by improving particle dispersion and contact between neighboring cellulose nanostructures (Csiszar et al., 2016).

Several strategies have been reported to improve CNF-based nanopaper, including water evaporation (Wang et al., 2019), NaOH post-treatment (Nan et al., 2017), spinning followed by stretching (Kafy et al., 2017), and hot-pressing (Österberg et al., 2013). Hot pressing or atmospheric heat treatment may induce thermal oxidation and yellowing of cellulose due to oxygen exposure and limited moisture (Matsuo et al., 2012).

Non-pressurized hot water vapor (NPHWV) treatment has recently been applied to BC pellicles to enhance mechanical performance (Rahmadiawan et al., 2024). However, this method differs from the present approach in material form and treatment state. NPHWV treats intact BC pellicles using vapor-phase heating, whereas this study applies boiling water (BW) treatment at 100 °C to a fully dispersed and sonicated BC powder suspension. This enables more uniform heat transfer, greater surface accessibility, and deeper molecular reorganization during nanopaper formation.

The distinct advantage of the present approach lies in applying BW treatment to the fully dispersed suspension before film formation. Ultrasonication initially disrupts BC powder aggregates and increases fibril accessibility, while subsequent heating in water provides greater molecular mobility for fibril rearrangement. Unlike treatments applied to intact pellicles or preformed films, this suspension-phase process allows the fibrils to reorganize before being immobilized during drying. Therefore, the method combines ultrasonic dispersion, aqueous thermal rearrangement, and drying-induced network consolidation in a single processing sequence.

Moreover, NPHWV relies on TEMPO-oxidized BC, which requires chemical reagents, increases environmental burden, and may alter native hydrogen-bonding sites through selective oxidation of primary hydroxyl groups (Park et al., 2024). In contrast, this work uses non-oxidized BC and avoiding chemical modification. Hence, preserving native hydroxyl functionalities. The process is simple, eco-friendly, and provides a clear platform to evaluate the effect of physical treatment alone on the structural and mechanical properties of BC nanopaper.

We hypothesize that BW treatment of sonicated BC-powder suspension can simultaneously improve the tensile strength and toughness of BC nanopaper without thermal oxidation. This study investigates the effect of BW treatment duration on the morphology, chemical structure, crystallinity, thermal stability, and tensile properties of BC nanopapers using FE-SEM, FTIR, XRD, thermogravimetric analysis, and tensile testing.

## 2 Materials and methods

### 2.1 Materials

BC pellicles were purchased from a small-scale industry in Padang, Indonesia. Briefly, a BC pellicle was obtained from the fermentation of glucose (4 g), acetic acid (5 mL), ammonium sulfate (4 g), coconut water mixture (1 L) by *Acetobacter xylinum* (50 mL) for 10 days. Sodium hydroxide (NaOH) and n-butyl alcohol were obtained from Brataco (Jakarta, Indonesia). Distilled water was used as a solvent.

### 2.2 Preparation of BC nanopaper

#### 2.2.1 BC powder preparation

**FIGURE 1:**
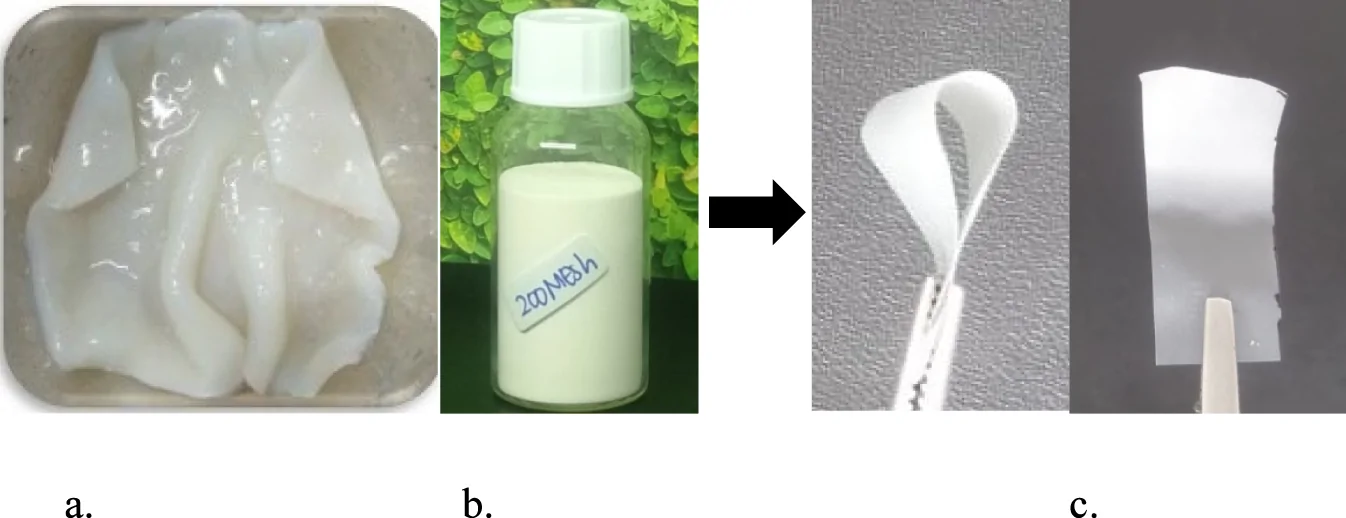
**(a)** BC pellicle as purchased, **(b)** BC powders of 75 μm in a bottle, **(c)** BC nanopaper.

A BC pellicle (Figure 1a) was purified by soaking in 1 L distilled water containing 10 g NaOH for 48 h to reach a final pH of 7. After the purification, it was further disintegrated using an electrical blender (Philips) at 12,000 rpm for 1 h. The resulting BC suspension was cast on a Teflon-coated frying pan and further dried with a drying oven (Universal Oven Memmert UN-55, Germany) at 50 °C for 24 h. Subsequently, the disintegrated, cast, and dried BC suspension was ground using an electric grinder (model SK Bean Grinder 800A) for 20–30 min at 36,000 rpm to obtain BC powder. Following the grinding process, the BC powder was passed consecutively through stainless-steel sieves with mesh sizes of 60, 100, and 200, from the largest to the smallest mesh size. The powder fraction that passed through the 200-mesh sieve was collected and denoted as PWDR. Based on the ASTM standard sieve opening, a 200-mesh sieve corresponds to an aperture size of approximately 74 μm; therefore, the PWDR sample was estimated to have a particle size of less than 74 μm. PWDR was used directly as a powdered comparative sample for XRD, FTIR, and thermogravimetric analyses and was not processed into nanopaper. A separate portion of the PWDR sample was used for the subsequent preparation of BC nanopapers.

#### 2.2.2 Samples without BW-treated suspension

One gram of PWDR was dispersed in 200 mL of distilled water and heated using a hotplate magnetic stirrer at 100 °C for 1 h. The suspension was then homogenized by a WiseTis homogenizer at 12,000 rpm for 30 min. The homogenization process of the BC suspension was continued using a 20 kHz ultrasonic cell crusher (Model SJIA-1200 W, Ningbo Yinzhou Sjia Lab Equipment Co., Ltd., Ningbo, China) at 1000 W for 1 h. The resulting sonicated BC suspension was cast onto a Teflon-coated frying pan and dried in an oven to produce the reference nanopaper, hereafter denoted as RAW.

#### 2.2.3 Samples from BW-treated suspension

The samples subjected to BW treatment, denoted as SUS, were prepared by heating the RAW BC suspension at 100 °C using a hotplate magnetic stirrer. The number following the SUS designation indicates the BW treatment duration: 2 h (SUS2), 4 h (SUS4), and 5 h (SUS5). The treated suspensions were subsequently cooled at room temperature for 24 h, cast onto a Teflon-coated frying pan, and dried in an oven to produce the corresponding nanopapers.

### 2.3 Characterization of BC nanopapers

The morphology, crystalline structure, chemical functional groups, thermal stability, and mechanical properties of BC nanopapers were characterized. Surface morphology was observed by FE-SEM using a JSM-6700F microscope (JEOL, Japan) at 1.5 kV for low magnification and a JIB 4610F FE-SEM microscope (JEOL, Japan) at 10 kV and 8 mA for high magnification. Freeze-dried samples were mounted on stubs using double-sided carbon tape and sputter-coated with platinum or carbon/gold prior to observation.

The apparent fibril diameter was quantified from the high-magnification FE-SEM micrograph using ImageJ software. The diameter values were reported as the mean ± standard deviation.

XRD patterns were recorded using a PANalytical X’Pert Pro diffractometer with CuKα radiation (λ = 0.154 nm) at 40 kV and 30 mA over 2θ = 5°–50°. The crystallinity index (CI) was calculated as:

$$CI=\frac{I_{002}-I_{am}}{I_{002}}x100$$

where I_002_ was the maximum crystalline peak intensity at around 2θ = 22.8° and I_am_ was the minimum intensity of the amorphous fraction between the 110 and 200 peak.

FTIR spectra were collected using a Frontier FTIR spectrometer (PerkinElmer, United States) from 4,000 to 600 cm^-1^ with 4 cm^-1^ resolution and 32 scans. Prior to analysis, nanopapers were dried at 50 °C for 24 h, conditioned at 50% RH for 48 h, ground, mixed with KBr, and pressed into pellets.

Thermal degradation was analyzed using a DTG-60 DTA-TG apparatus (Shimadzu, Japan). Approximately 10 mg of each sample was heated from room temperature to 600 °C under nitrogen flow of 50 mL/min at 10 °C/min.

Tensile properties were measured using a Com-Ten 95T Series testing machine with a 25 kg load cell. Samples were cut according to ASTM D638 Type V, conditioned at 50% ± 5% RH for 48 h, and tested at room temperature and approximately 75% RH with a crosshead speed of 5 mm/min. The average thickness of all nanopaper samples was approximately 54 μm.

Tensile strength, tensile modulus, elongation at break, and toughness, calculated from the area under the stress-strain curve (Wang et al., 2018b), were determined from five replicates. The tensile data were statistically analyzed using one-way analysis of variance (ANOVA), and differences were considered significant at (p < 0.05).

## 3 Results and discussion

The different formats of BC pellicle, BC powder, and the resulting nanopaper as shown in Figure 1. The as-purchased BC pellicle (Figure 1a) appeared as a translucent white hydrogel with a soft and highly hydrated structure. After drying, grinding, and sieving, the BC was converted into a fine white powder with a particle size below 75 μm (Figure 1b). The powder was subsequently processed into nanopaper (Figure 1c), which exhibited a smooth, uniform, and defect-free appearance. The nanopaper could be folded without visible cracking, indicating good flexibility and structural integrity. These observations demonstrate that BC powder can serve as an effective precursor for the fabrication of flexible nanopaper through a simple and environmentally friendly process.

### 3.1 Surface morphologies of nanopapers

**FIGURE 2:**
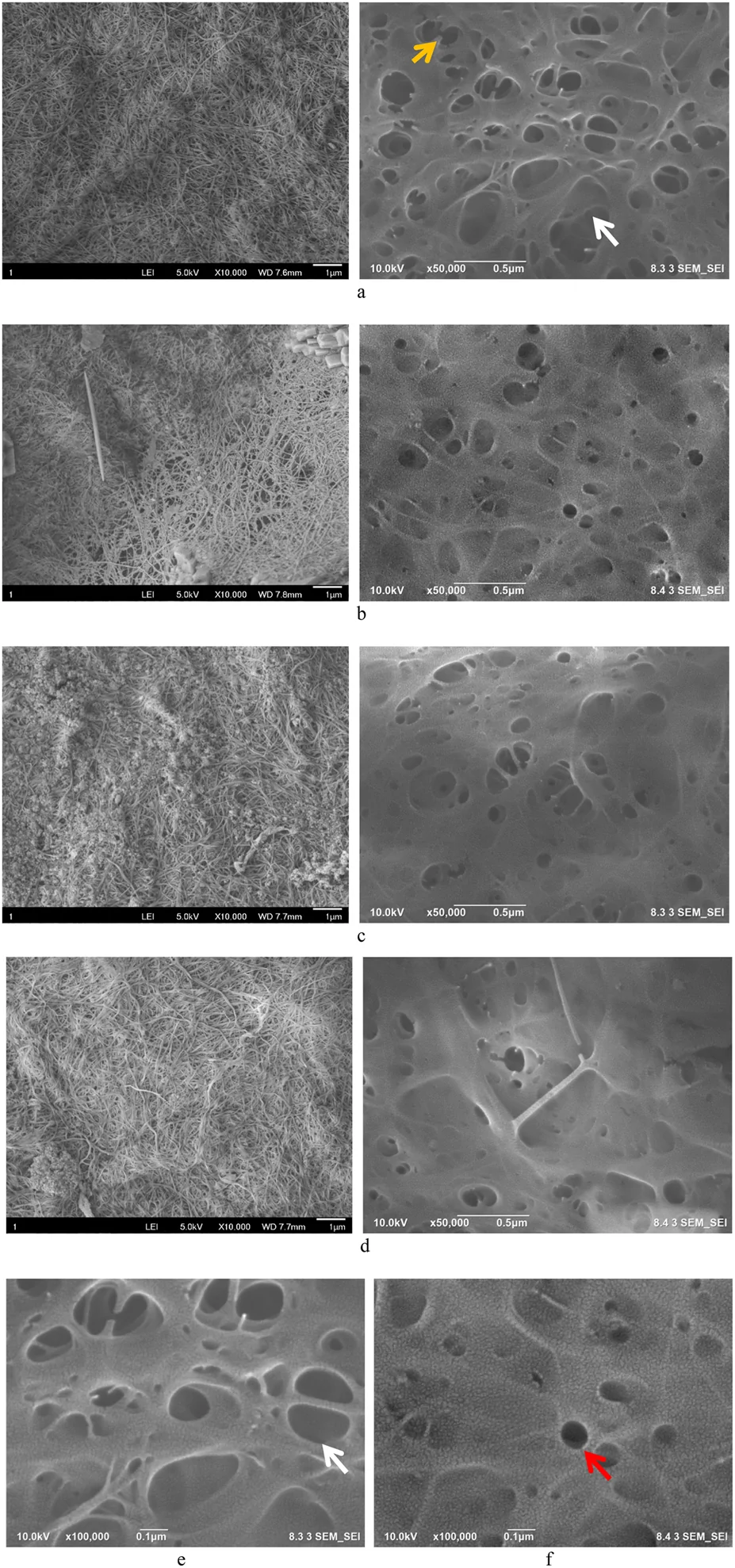
Representative electron micrographs of BC nanopaper surfaces at 10k in LEI mode (left) and 50k in SEI mode (right) of **(a)** RAW, **(b)** SUS2, **(c)** SUS4, **(d)** SUS5. The surface appearance of RAW **(e)** and SUS4 **(f)** nanopaper at 100k magnification in SEI mode. The arrows with orange, white, and red mark the fractured sections, micro-sized pores, and nano-sized pores, respectively.

To investigate the morphologies of the nanopapers, electron microscopy was performed on nanopapers fabricated without (RAW) and with (SUS) boiling water treatments for 2 (SUS2), 4 (SUS4), and 5 (SUS5) hours. Electron micrographs of the surface for all nanopapers are presented in Figure 2. The surface appearances of all samples at 10k magnification are similar, showing distinct interwoven BC fibers (Figures 2a-d, left). The observations are consistent with other BC preparations without boiling for a prolonged duration (Basu et al., 2018).

Quantitative analysis of the high-magnification FE-SEM micrograph using ImageJ showed that the apparent fibril diameter ranged from 26 to 116 nm, with an average value of 53.7 ± 19.2 nm. These results confirm that the micrometer-sized BC powder particles were composed of an interconnected nanoscale fibrillar network, supporting the use of the term “nanopaper.”

At 50k magnification, the RAW sample (Figure 2a, right) shows a higher number of micro-sized pores (marked with a white arrow) than the SUS nanopaper. This sample also displays fractured sections (orange arrow). After the treatment of the sonicated BC suspension in boiling water, the dimension and quantity of the pores in the treated nanopaper appear to decrease within the field of view (Figures 2b,c, right). SUS4 nanopaper (Figure 2c, right) displayed more nano-sized pores (red arrow) and had lower numbers of these defects than the non-treated nanopaper (Figure 2a, right). The number of pores appears to be reduced and is inversely related to the treatment duration.

The thermal energy supplied by the boiling water may facilitate the retention of a compact cellulose structure with a higher fraction of highly ordered crystalline regions (Nakagaito et al., 2010). Heating may enhance water mobility within the interfibrillar spaces of the BC network, facilitating water redistribution during treatment. During subsequent film formation and drying, water removal and capillary forces may promote closer contact and rearrangement of the cellulose fibrils. This closer fibril association may facilitate the formation of interfibrillar hydrogen-bonded networks, reducing void dimensions and producing a denser nanopaper microstructure (Österberg et al., 2013; Vasconcellos and Farinas, 2018). Therefore, boiling-water treatment may provide an alternative approach for preparing compact BC films with reduced porosity (Sun, 2008).

### 3.2 Crystallinity of BC powder and BC nanopaper

**FIGURE 3:**
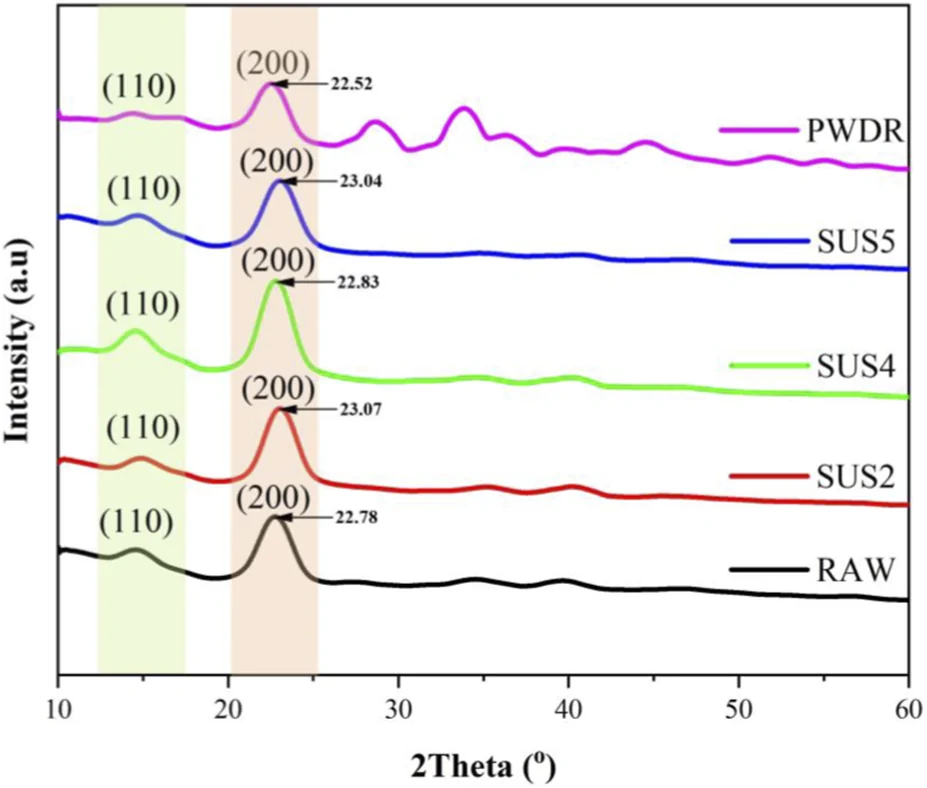
XRD diffractogram of BC powders and BC nanopaper treated without and with the BW treatment.

The XRD patterns of BC powder and BC nanopapers are shown in Figure 3. The main peaks at 2θ ≈ 14.7°, 16.5°, and 22.8° correspond to the (1–10), (110), and (200) planes of cellulose I crystals (Xiong et al., 2014). Overall, treated nanopapers showed higher structural order than the untreated sample (Supplementary Table S1), likely due to more effective removal of amorphous regions during boiling-water treatment (Sun et al., 2015). Compared with PWDR, ultrasonicated BC suspensions (RAW and SUS samples) exhibited sharper (200) peaks, indicating improved crystalline regularity (Zhang et al., 2012).

The (200) peak shifted slightly from 22.5° in PWDR to 22.8° in RAW. According to Bragg’s law, this shift toward a higher diffraction angle corresponds to a decrease in the interplanar spacing from approximately 3.95 to 3.90 Å. This slight decrease may reflect a more compact arrangement of the cellulose crystalline planes following suspension processing, film formation, and drying. Nevertheless, the preservation of the characteristic cellulose I reflections and the absence of new diffraction peaks associated with other cellulose polymorphs suggest that the cellulose I structure was retained (Lin et al., 2020), (French, 2014).

The crystallinity index (CI) varied with boiling duration. RAW showed a CI of 63.13%, while boiling increased the CI, reaching the highest value in SUS4 at 68.73%. This indicates that 4 h boiling promoted cellulose chain alignment and packing. In contrast, SUS2 and SUS5 showed lower CI values of 66.46% and 63.75%, respectively, indicating that a shorter treatment duration was insufficient to achieve maximum structural ordering, whereas prolonged boiling did not provide further improvement. This trend was consistent with the FTIR results, which indicated corresponding changes in hydrogen-bonding interactions and cellulose-chain organization, as will be discussed in Section 3.3.

PWDR exhibited the lowest CI of 39.91%, probably due to disruption of crystalline domains during drying and powdering, which increased the amorphous fraction. It also showed additional weak peaks, possibly related to residual components or structural variations introduced during drying. After treatment, the interlayer spacing d of the (1–10) and (200) planes decreased and the peak positions shifted to higher angles (Supplementary Table S1), indicating a more compact crystalline arrangement (Ju et al., 2015). For example, 2 h boiling reduced the (200) spacing from 3.94 to 3.86 Å and increased the CI by 3.5%, confirming that boiling-water treatment promoted cellulose chain reordering. Similar structural reordering of BC nanofibers after mechanical treatment was also reported in our previous work (Abral et al., 2018). Normalized XRD patterns in Supplementary Figure S1 further show weak diffraction peaks besides the main cellulose I reflections, which may originate from structural changes during defibrillation and drying.

### 3.3 Functional group analyses upon BW treatment

The effect of BW treatment on the functional groups of BC nanopaper analyzed using FTIR is shown in Supplementary Figures S2a–e. Supplementary Figure S2a presents the FTIR spectra of all samples in the range of 4,000–250 cm^-1^. Changes in band position, sharpness, and intensity indicate structural rearrangement induced by mechanical and BW treatments. The broad band at around 3,326 cm^-1^ corresponds to O–H stretching and hydrogen bonding of hydroxyl groups (Ridzuan et al., 2016). After treatment, this peak shifted from 3,326 cm^-1^ in RAW to 3,320 cm^-1^ in SUS2, suggesting changes in intermolecular and intramolecular hydrogen-bonding interactions. A shift of the O–H band toward a lower wavenumber is generally associated with stronger or more extensive hydrogen-bonding interactions, which may facilitate closer cellulose-chain association and structural ordering. The sharper O–H band observed after BW treatment also suggests a more uniform hydrogen-bonding environment and cellulose-chain reorganization (Olsson and Salmén, 2004). These changes are consistent with the variation in crystallinity index obtained from XRD. In particular, the higher CI of SUS4 suggests more effective hydrogen-bonded chain packing, whereas the lower CI values of SUS2 and SUS5 indicate less optimal structural ordering.

The absorbance ratio between the O–H stretching band at approximately 3,326 cm^-1^ and the CH_2_-wagging band at approximately 1,320 cm^-1^ was used to evaluate changes in hydrogen-bonding interactions relative to the ordered cellulose structure (Supplementary Table S1). The CH_2_-wagging vibration is associated with conformational ordering in cellulose; therefore, variations in this ratio provide complementary evidence of changes in chain organization.

The FTIR ratio followed the same general trend as the XRD-derived CI, supporting the conclusion that BW treatment altered the degree of cellulose-chain packing without changing the principal chemical structure of BC. The increased transmittance of the O–H band may also indicate a reduced relative contribution from accessible hydroxyl groups or differences in hydrogen-bonding environments (Soo et al., 2021), consistent with the denser microstructure of the treated nanopapers shown in Figure 2.

The band at approximately 2,890 cm^-1^ is assigned to C–H stretching vibration. RAW showed stronger intensity, likely due to greater structural heterogeneity, disoriented crystals, and nonuniform strain generated by mechanical treatment (Abral et al., 2020). After BW treatment, the peak intensity decreased, indicating reordering of the cellulose structure. The bands at 1,634–1,651 cm^-1^ correspond to O–H bending of absorbed water (Thiripura Sundari and Ramesh, 2012), while their shift to higher wavenumber may reflect polymer swelling caused by water molecule occupation (Velazquez et al., 2003).

Supplementary Figure S2e shows the enlarged spectra in the 1,400–1,250 cm^-1^ region. The bands observed at approximately 1,323–1,342 cm^-1^ were associated with CH_2_-wagging and C–H-bending vibrations of cellulose, which are sensitive to chain conformation and structural organization (Chen et al., 2015). PWDR exhibited a band at approximately 1,323 cm^-1^, whereas RAW, SUS2, SUS4, and SUS5 showed bands at approximately 1,335, 1,342, 1,334, and 1,338 cm^-1^, respectively. The differences in band position among the samples indicate changes in the local conformational environment and packing of the cellulose chains after suspension processing and BW treatment.

The A3326/A1320 ratio represents the relative absorbance of the O–H stretching band to that of the CH_2_-wagging/C–H-bending band and was used to assess changes in the hydrogen-bonding environment relative to cellulose-chain organization. The ratios were 1.022, 0.956, 0.989, 0.954, and 0.989 for RAW, SUS2, SUS4, SUS5, and PWDR, respectively (Supplementary Table S1). Among the BW-treated samples, SUS4 exhibited a higher ratio than SUS2 and SUS5, consistent with its higher crystallinity index and suggesting more effective hydrogen-bonded chain organization after 4 h of treatment.

However, RAW showed the highest ratio, while PWDR exhibited the same value as SUS4 despite having substantially lower crystallinity. Therefore, the A3326/A1320 ratio should not be considered a direct measure of crystallinity because it may also be influenced by hydroxyl-group accessibility, retained water, and the local hydrogen-bonding environment. Nevertheless, the FTIR results provide complementary evidence that BW treatment altered cellulose-chain interactions and structural organization, in agreement with the XRD results.

### 3.4 TG and DTG analysis

Supplementary Figure S5 shows the thermal properties of BC powder and nanopapers prepared without and with BW treatment for 2, 4, and 5 h. All samples exhibited an initial weight loss at 60 °C–150 °C due to evaporation of absorbed water (Supplementary Figure S5a). The RAW nanopaper showed a higher evaporation rate than the BW-treated samples, likely because of its higher porosity. After 5 h boiling, SUS5 showed only 10.3% weight loss, 20.2% lower than RAW, indicating reduced water retention in the treated nanopaper (Mahardika et al., 2018).

Further heating caused a major weight loss at 240 °C–350 °C, corresponding to cellulose decomposition (Sya et al., 2018; Asrofi et al., 2018). The BC powder showed the lowest maximum decomposition temperature (T_m_) of 260.5 °C, probably due to its highly disordered cellulose structure and lowest CI value of 39.91% (Supplementary Table S1). A third weight loss above 350 °C may be associated with thermal-oxidative decomposition of char residues (Ilyas et al., 2018).

As shown in Supplementary Figure S5b, BW-treated nanopapers generally exhibited higher thermal resistance than RAW. For example, 4 h boiling increased T_m_ from 302.7 °C for RAW to 307.6 °C for SUS4. Among the treated samples, SUS4 exhibited the highest T_m_, corresponding to an increase of 4.9 °C relative to RAW. This improvement is attributed to a more compact network structure (Figure 2f), lower porosity, and higher crystallinity (Supplementary Table S1), requiring more energy for decomposition. Similar enhancement in nanopaper thermal properties after water evaporation treatment has also been reported (Wang et al., 2019).

In contrast, SUS2 exhibited a lower T_m_ of 291.1 °C, indicating that a shorter BW treatment did not produce the same degree of thermally stable structural reorganization as the 4 h treatment. SUS5 showed a T_m_ of 302.2 °C, which was close to that of RAW but lower than that of SUS4. This result suggests that prolonged boiling did not provide additional thermal stabilization. The lower CI of SUS5 compared with SUS4 may indicate partial disruption or reduced ordering of crystalline regions after extended treatment, thereby offsetting the beneficial effect of network densification.

At approximately 550 °C, the nanopaper samples retained about 33%–37% residual mass, whereas PWDR retained approximately 47%–48%. The higher residue of PWDR, despite its lower T_m_, indicates that maximum degradation temperature and char yield describe different aspects of thermal behavior and should not be interpreted as directly proportional. Overall, the results demonstrate that an optimum BW treatment duration of 4 h enhanced the thermal resistance of the BC nanopaper, whereas shorter or prolonged treatment produced less pronounced improvements. Thus, appropriate BW treatment can improve the mechanical performance and thermal resistance of BC nanopaper without chemical modification.

### 3.5 Tensile properties

Figure 3a shows the engineering tensile stress–strain curves of nanopapers without and with BW treatment for 2, 4, and 5 h. The RAW sample exhibited a mainly linear elastic response without a clear yield point or strain-hardening region, indicating less ductile behavior with limited post-elastic deformation. This behavior may be related to the higher fraction of large pores (Figure 2a) and rigid nanofibers (Sehaqui et al., 2011), which lowers the energy required for crack propagation compared with cellulose chain movement (Meng et al., 2017). In contrast, BW-treated nanopapers showed a plastic deformation region after the elastic stage, as seen in the inset of Figure 3a, suggesting improved nanofiber mobility.

Similar enhancements have been reported for ultrasonically treated BC nanopapers. Batishcheva et al. found that ultrasonication increased the tensile strength of BC nanopaper from approximately 78–81 MPa to 121–134 MPa (Batishcheva et al., 2022). Somvanshi and Gope also reported improved tensile strength of PVA/cellulose nanofiber films from 70.13 to 80.47 MPa after ultrasonication, due to nanofiber deagglomeration, better dispersion, and more efficient stress transfer (Somvanshi and Gope, 2021).

The improved chain mobility is consistent with structural relaxation or expansion, as supported by XRD results and FE-SEM morphology (Figures 2e,f). Treated nanopapers also displayed strain hardening, where stress increased with strain in the second region. This indicates that crack propagation was more strongly hindered in the treated nanopapers than in RAW.

**FIGURE 4:**
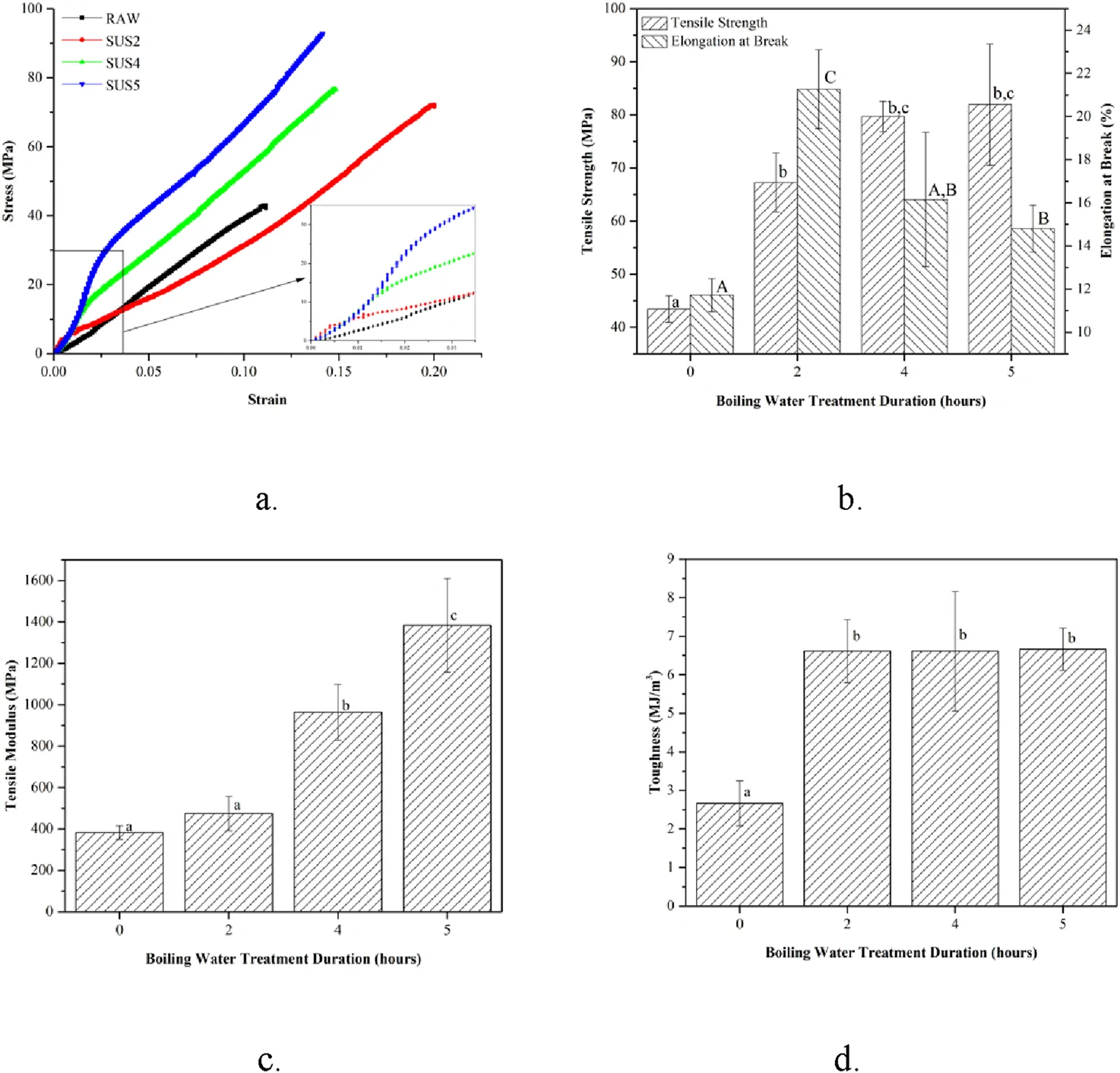
The tensile stress-strain curve for each tested BC nanopaper **(a)**; the average values of TS and EB **(b)**, TM **(c)**, and TN **(d)** for non-boiled and boiled BC nanopapers.

ANOVA confirmed that BW treatment significantly affected tensile strength (TS), tensile modulus (TM), elongation at break (EB), and toughness (TN) (p < 0.05; Figures 4b–d). RAW showed the lowest TS, TM, EB, and TN values of 43.4 MPa, 382.9 MPa, 11.7%, and 2.7 MJ/m^3^, respectively. The highest TS and TM were obtained for SUS5, reaching 81.9 MPa and 1,383.5 MPa, with a TN of 6.7 MJ/m^3^. Compared with RAW, SUS5 increased TS, TM, and TN by 89%, 261%, and 148%, respectively.

Compared with previous studies, this method provides competitive performance using a simple and chemical-free process. Du et al. reported cellulose nanopaper from paper mill sludge with TS of 106.4 MPa and TN of 6.62 MJ/m^3^ using a chemically intensive process (Du et al., 2020), while Wang et al. achieved TS of 255 MPa and TN of 19.7 MJ/m^3^ by incorporating lignin into tobacco stalk-derived cellulose (Wang et al., 2018b). Although these values are higher, they require chemical modification or more complex processing. In contrast, SUS5 achieved TS of 81.9 MPa and TN of 6.7 MJ/m^3^ using only BW treatment, demonstrating a simpler, lower-cost, and more environmentally friendly route for producing BC nanopaper with improved mechanical performance.

## 4 Conclusion

This work has successfully fabricated BC nanopaper with improved tensile strength and toughness from dry BC powders obtained from BC pellicle using an environmentally friendly method. The prolonged boiling water treatment of suspended BC powder suspension and subsequent sonication improved the strength and toughness of the BC nanopaper simultaneously. The BC nanopaper fabricated using boiled BC powder suspension had more plastic deformation and a higher value of crystallinity index than the non-boiled one. The higher temperature of the boiling water expanded the polymer structure, decreasing porosity fractions. These findings demonstrate that BW treatment of sonicated BC suspension is an effective chemical-free strategy to simultaneously enhance the strength and toughness of BC nanopaper, providing a sustainable pathway for the development of high-performance cellulose-based materials for packaging, filtration, and flexible functional applications.

## Data Availability

The original contributions presented in the study are included in the article/Supplementary Material, further inquiries can be directed to the corresponding author.
