# Supplementary material for: Eco-friendly fabrication of bacterial cellulose nanopaper with enhanced strength and toughness from a powder suspension
Source: Front Bioeng Biotechnol. 2026 Aug 25;14:1923616. doi: 10.3389/fbioe.2026.1923616 (PMC13550159; doi:10.3389/fbioe.2026.1923616)
Supplement: Supplementary file 1 [file Supplementaryfile1.docx]

**Supplementary Information**

Eco-Friendly Fabrication of Strong and Tough Nanopaper from Bacterial Cellulose Powder Suspension

Dieter Rahmadiawan^1,2^, Hairul Abral^3^*, Muhammad Irfan^3^, Fadli Hafizulhaq^3^,

Sundaravadanam Vishnu Vadanan^4^, Sierin Lim^4^, Shih-Chen Shi^1^

^1^ Department of Mechanical Engineering, National Cheng Kung University (NCKU), Tainan 70101, Taiwan

^2^ Department of Mechanical Engineering, Universitas Negeri Padang, 25173 Padang, Sumatera Barat, Indonesia

^3^ Laboratory of Nanoscience and Technology, Department of Mechanical Engineering, Andalas University, Padang 25163, Indonesia

^4^ School of Chemical and Biomedical Engineering, Nanyang Technological University, 70 Nanyang Dr., Block N1.3, Singapore 637457, Singapore

^*^Corresponding author: habral@yahoo.com

**Table S1:** The crystallinity index (CI) from XRD, *d*-spacing, absorbance ratio between (A3326/A1320) cm^-1^, of all samples

| Sample | 2θ of (200) plane | CI (%) at 2θ = 22.8° | *d*-spacing [Å] of the lattice plane for | | (A3326/A1320) cm^-1^ from FTIR |
| --- | --- | --- | --- | --- | --- |
|  |  |  | (1-10) | (200) |  |
| RAW | 22.78 | 63.13 | 6.08 | 3.94 | 1.022 |
| SUS2 | 23.07 | 66.46 | 5.91 | 3.86 | 0.956 |
| SUS4 | 22.83 | 68.73 | 5.99 | 3.89 | 0.989 |
| SUS5 | 23.04 | 63.75 | 5.83 | 3.86 | 0.954 |
| PWDR | 22.52 | 39.91 | 5.29 | 3.91 | 0.989 |

**Crystallinity of bacterial cellulose films- XRD**

Figure S1: X-ray diffractogram of various bacterial cellulose films, new peaks observed apart from the characteristic BC peaks at 14°, 16° and 22°.

|  |  |
| --- | --- |
| a. | b. |
|  |  |
| c. | d. |
|  | |
| e. | |

Figure S2: FTIR curves of the bacterial cellulose nanopaper without and with treatment; (a) the full spectrum from 4000-250 cm^-1^, (b-e) sections of the spectrum expanded to show changes in peaks related to specific functional groups.

**Chemical structure of dried bacterial cellulose films- FTIR**

Figure S3: Normalized IR spectra of bacterial cellulose films measured in reflection mode. The spectra were vertically offset for clarity and comparison among RAW, SUS2, SUS4, and SUS5.

Figure S4: IR spectrum of various bacterial cellulose films in transmission measuring mode.

Certain crystal structures are present on the surface of treated BC films. X-ray diffractograms display new crystal peaks apart from the existing characteristic BC peaks of 14°, 16° and 22°. The presence of the additional peaks could be due to the BC source or the different treatments applied for defibrillation and drying. The IR spectra show retention of chemical structure despite the defibrillation and drying process.

|  |  |
| --- | --- |
| a. | b. |

Figure S5: Thermal Properties of BC Nanopapers (a) TG, (b) DTG.
